# A Rare Manifestation of Asymptomatic Ebstein's Anomaly with Tricuspid Valve Endocarditis

**DOI:** 10.1155/2017/7630915

**Published:** 2017-10-25

**Authors:** Carmel Moazez, Vicken Zeitjian, Christian Breburda, Ranjini Roy

**Affiliations:** Maricopa Integrated Health System, 2601 E. Roosevelt St., Phoenix, AZ 85008, USA

## Abstract

Ebstein's anomaly is a rare congenital heart disease that presents with apical displacement of the septal and posterior leaflets of the tricuspid valve. It has a wide spectrum of clinical presentations and has been shown to manifest itself any time from birth to adulthood. Our patient is a 43-year-old male with a history of intravenous heroin abuse who presented to the emergency department with worsening shortness of breath and lower extremity edema. He denied any prior cardiac history. A transthoracic echo showed normal left ventricular function, but a large 2.2 × 2.1 cm echodensity on the septal leaflet of the tricuspid valve consistent with vegetation with severe tricuspid regurgitation and probable leaflet perforation. It also demonstrated severe right heart enlargement with atrialization of the right ventricle and apical displacement of the tricuspid valve consistent with Ebstein's anomaly. This is a rare case of an adult who presented with asymptomatic Ebstein's anomaly. There have been few reports of tricuspid valve endocarditis with Ebstein's anomaly in the literature. To our knowledge, this represents the fifth reported case of a new diagnosis of Ebstein's anomaly in the setting of endocarditis and the second case of Ebstein's anomaly and endocarditis in an intravenous drug abuser.

## 1. Introduction

Ebstein's anomaly is a rare congenital heart disease that presents with apical displacement of the septal and posterior leaflets of the tricuspid valve. The incidence of Ebstein's anomaly is about 1 per 200,000 live births [1]. It has a wide spectrum of clinical presentations and has been shown to manifest itself any time from birth to adulthood. When it presents in adulthood, it will typically present with symptomatic arrhythmia, usually tachycardia, or right or left sided heart failure [2].

On exam, patients will present with murmur of tricuspid regurgitation, widely split first heart sound, and jugular venous distention. Forty-seven percent of adult patients with Ebstein's anomaly have cyanosis due to increased right atrial pressure from tricuspid regurgitation. Electrocardiogram findings in Ebstein's anomaly include tall and peaked p waves, widened QRS with or without right bundle branch block, and qR pattern in leads V1–V4. Transthoracic echocardiogram is the test of choice for diagnosing Ebstein's anomaly [3].

It has been thought that Ebstein's anomaly with tricuspid regurgitation can predispose a person to tricuspid valve endocarditis, but very few cases in the literature have shown this. Rarely will Ebstein's anomaly remain undetected into late adulthood and present as an incidental finding [2].

## 2. Case Presentation

The patient is a 43-year-old male with a history of intravenous heroin abuse who presented to the emergency department with worsening shortness of breath and lower extremity edema. He had shortness of breath and leg swelling for one month, which worsened in the past week. He also complained of fever, chills, chronic cough, pleuritic chest pain, and abdominal pain. Previously he had been able to walk indefinitely, but at this time he could only walk a few steps before becoming fatigued. He denied taking any medications and denied drug allergies. He denied history of surgeries. He had never been hospitalized. He denied cardiac history and ever having been “blue” as a child. His mother was an alcoholic and likely used lithium, which he believed were continued during pregnancy. He had lost contact with his biological mother. He had a 30-pack year smoking history and admitted to using intravenous heroin just prior to admission. He reused needles and tested negative for HIV and hepatitis one year prior to admission.

Exam on presentation was significant for a man in moderate distress. He had a 2/6 holosystolic heart murmur heard best at the left sternal border and apex, which increased with respiration. He also had right upper quadrant tenderness, Murphy's sign positive, and 3+ pitting edema in the lower extremities. No splinter hemorrhages, Osler nodes, or Janeway lesions were visualized.

The patient was hypotensive, hypoxic, tachycardic, and tachypneic on admission. Electrocardiogram was consistent with right bundle branch block with QRS duration 124 ms. Pertinent labs included white blood cell count 33.6 k/uL, brain natriuretic peptide (BNP) 3,517 pg/mL, hemoglobin 10.5 g/dL with MCV 79.9 fl, HIV screening test negative, hepatitis C antibody positive, and urine drug screen positive for barbiturates.

**Figure 1:**
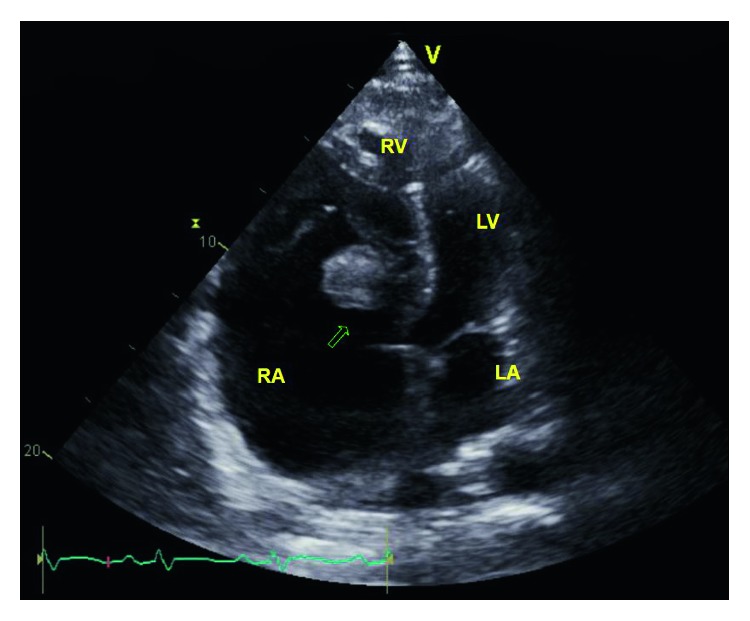
Apical view showing the tricuspid valve vegetation and apical displacement of the tricuspid valve.

**Figure 5:**
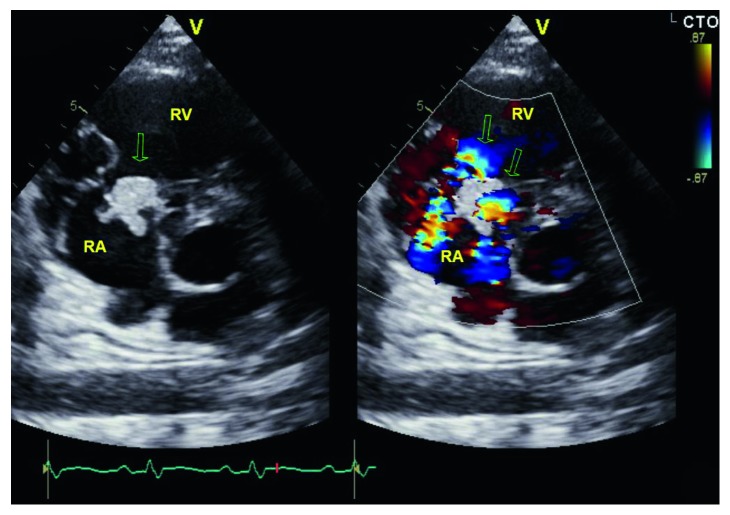
Parasternal short access view color flow Doppler showing the tricuspid regurgitation jet off axis and vegetation.

He was admitted to the medical intensive care unit and started on vancomycin and zosyn for endocarditis. Blood cultures were drawn prior to starting antibiotics, which grew *Streptococcus oralis*. A transthoracic echocardiogram showed normal left ventricular function with ejection fraction 55–60%, a large 2.2 × 2.1 cm echodensity on the septal leaflet of the tricuspid valve consistent with a vegetation with severe tricuspid regurgitation, and probable leaflet perforation. Figures 1()–3() show different views of the large vegetation that was visualized. Echocardiogram also showed severe right heart enlargement with atrialization of the right ventricle and apical displacement of the tricuspid valve consistent with Ebstein's anomaly, which can be seen in Figures 1() and 4(). Interatrial septum appeared intact with color flow Doppler, which can be visualized in Figure 5(). A computerized tomography (CT) chest scan showed septic pulmonary emboli, and he was started on intravenous heparin. The patient was stabilized and transferred to a higher-level care center for tricuspid valve repair.

## 3. Discussion

This is a rare case of an adult who presented with asymptomatic Ebstein's anomaly. To our knowledge, this represents the fifth reported case of a new diagnosis of Ebstein's anomaly in the setting of endocarditis and the second case of Ebstein's anomaly and endocarditis in an intravenous drug abuser [2, 4, 5]. The other case of Ebstein's anomaly and endocarditis in an intravenous drug abuser was in a 19-year-old patient who presented much younger than our patient [4]. The other two cases were pacemaker endocarditis [2, 6]. Our patient presented with endocarditis secondary to intravenous drug abuse, and Ebstein's anomaly was an incidental finding on transthoracic echocardiogram. His baseline anatomical abnormality of his tricuspid valve from his undiagnosed Ebstein's anomaly may have contributed to the development of his endocarditis.

Two-dimensional transthoracic echocardiography is the diagnostic test of choice for Ebstein's anomaly. Typical findings include apical displacement of the proximal attachment of the septal and posterior tricuspid valve leaflets greater than 8 mm/m^2^ body surface area or 1.2 cm in length, redundancy and fenestrations of the anterior tricuspid valve leaflet, and dilation of the anatomic tricuspid valve annulus at the atrioventricular junction [2, 3, 7]. These findings will cause decreased function of the right ventricle, dilation of the right atrium, and dilation of the right ventricle in addition to atrial and ventricular arrhythmias [8].

Fifty percent of patients who present in adulthood will have cyanosis, but this patient had no history of cardiac issues or cyanosis. Very rarely will a patient with Ebstein's anomaly survive into adulthood with no symptoms. Ebstein's anomaly has a wide spectrum of clinical presentation, which can range from death in a neonate to mild symptoms in an elderly person. Elderly patients who are found to have Ebstein's anomaly usually have milder forms and a better overall prognosis. Further, studies have shown that elderly patients will usually present with arrhythmias, likely supraventricular tachycardia. Paroxysmal supraventricular arrhythmias are a common cause of New York Heart Association (NYHA) functional class deterioration [7]. NYHA class I and II patients can be managed medically, while class III and IV will likely require surgical intervention [8]. Patients can also present with sudden cardiac death related to a conduction abnormality causing atrial fibrillation with preexcitation [3].

It is known that intravenous drug abuse predisposes patients to tricuspid valve endocarditis, but it is unknown whether Ebstein's anomaly also predisposes a patient to tricuspid valve endocarditis [4]. It is also thought that Ebstein's anomaly with tricuspid regurgitation predisposes patients to infective endocarditis [2]. More cases will need to be studied to determine this risk.

## 4. Conclusion

Ebstein's anomaly is a rare congenital anomaly that very rarely remains undetected into late adulthood. Our case describes a 43-year-old male with a history of intravenous heroin abuse found to have tricuspid valve endocarditis with an incidental finding of Ebstein's anomaly. There have been few reported cases of tricuspid valve endocarditis with Ebstein's anomaly in the literature. To our knowledge, this represents the fifth reported case of Ebstein's anomaly in the setting of endocarditis and the second case of Ebstein's anomaly and endocarditis in an intravenous drug abuser.

## Figures and Tables

**Figure 2:**
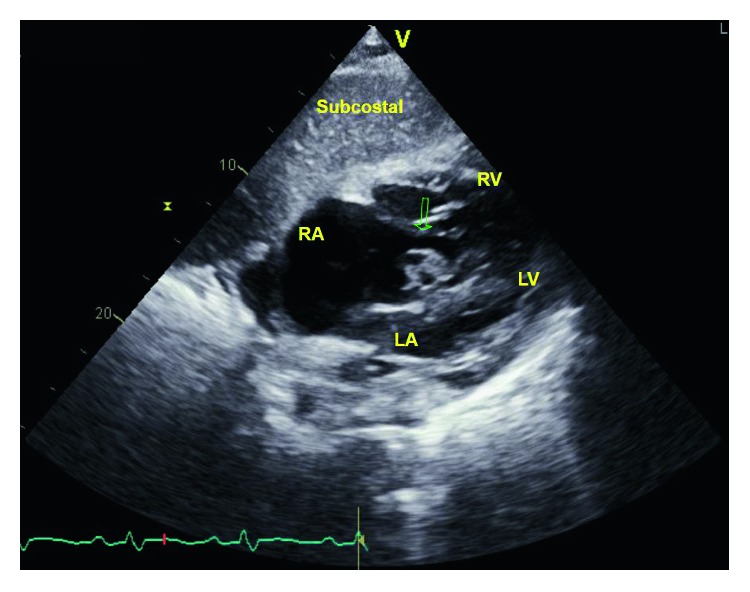
Subcostal view showing the tricuspid valve vegetation.

**Figure 3:**
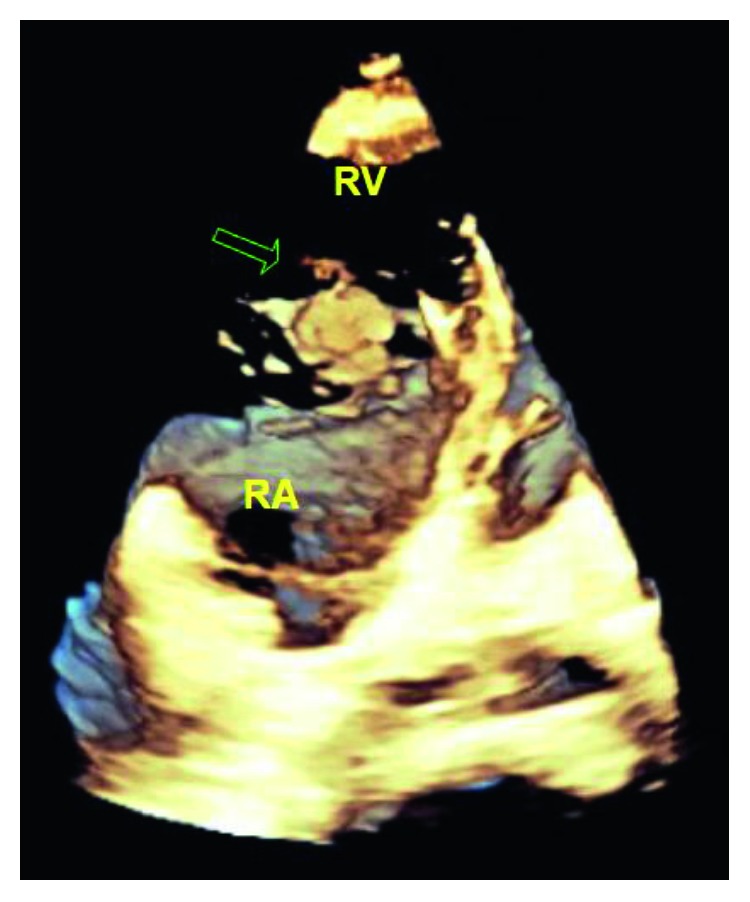
Three-dimensional image showing the tricuspid valve vegetation.

**Figure 4:**
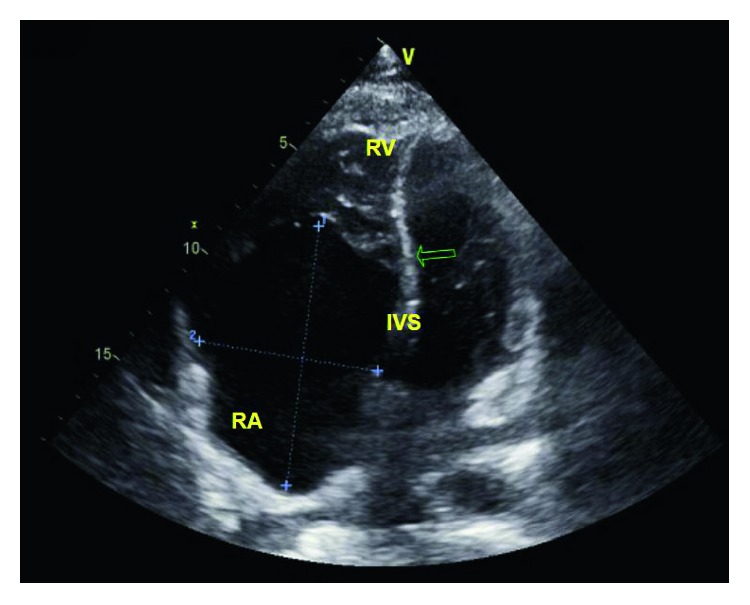
Apical 4 view showing apical displacement of the tricuspid valve leaflet causing atrialization of the right ventricle. This is diagnostic of Ebstein's anomaly.
